# Atrial conduction time, and left atrial mechanical and electromechanical functions in patients with polycystic ovary syndrome: interatrial conduction delay

**DOI:** 10.5830/CVJA-2015-046

**Published:** 2015

**Authors:** Emine Gazi, Ahmet Temiz, Burak Altun, Ahmet Barutcu, Yucel Colkesen, Meryem Gencer, Ayse Nur Gungor, Servet Hacivelioglu, Ahmet Uysal, Volkan Hanci

**Affiliations:** Department of Cardiology, Canakkale Onsekiz Mart University, Canakkale, Turkey; Department of Cardiology, Canakkale Onsekiz Mart University, Canakkale, Turkey; Department of Cardiology, Canakkale Onsekiz Mart University, Canakkale, Turkey; Department of Cardiology, Canakkale Onsekiz Mart University, Canakkale, Turkey; Department of Cardiology, Canakkale Onsekiz Mart University, Canakkale, Turkey; Department of Obstetrics and Gynecology, Canakkale Onsekiz Mart University, Canakkale, Turkey; Department of Obstetrics and Gynecology, Canakkale Onsekiz Mart University, Canakkale, Turkey; Department of Obstetrics and Gynecology, Canakkale Onsekiz Mart University, Canakkale, Turkey; Department of Obstetrics and Gynecology, Canakkale Onsekiz Mart University, Canakkale, Turkey; Department of Anesthesiology and Reanimation, Canakkale Onsekiz Mart University, Canakkale, Turkey

**Keywords:** electromechanical delay, polycystic ovary syndrome, P-wave dispersion, interatrial conduction

## Abstract

**Background:**

Polycystic ovary syndrome (PCOS) is one of the most common endocrine disorders of women during the reproductive period. Cardiovascular risk factors are more frequent in patients with PCOS. We aimed to investigate the P-wave dispersion (Pd), inter- and intra-atrial conduction time and mechanical functions of the left atrium (LA) in patients with PCOS.

**Methods:**

Forty-eight patients with PCOS and 38 normal healthy women were enrolled in this study. A 12-lead surface electrocardiogram was used to evaluate Pd. Left ventricular (LV) functions were measured using conventional and tissue Doppler imaging (TDI) methods. Inter- and intra-atrial conduction times were measured by TDI. LA volumes were measured echocardiographically with the biplane area–length method from the apical four-chamber view.

**Results:**

Heart rate (82.02 ± 13.15 vs 74.24 ± 11.02 bpm, *p* = 0.014) and Pd were significantly increased in the PCOS patients [27 ± 5 vs 24 ± 6 ms, *p* = 0.035]. Transmitral E/A ratio was significantly lower in the PCOS patients than in the controls (1.5 ± 0.3 vs 1.7 ± 0.4 m/s, *p* = 0.023). Passive emptying volume (12.54 ± 4.39 vs 15.28 ± 3.85 ml/m^2^, *p* = 0.004) and passive emptying fraction [54.4 (21–69) vs 59.1% (28–74), *p* = 0.008] were significantly decreased in PCOS patients. Total emptying volume was significantly decreased (17.9 ± 5.49 vs 20.67 ± 4.29 ml/m2, *p* = 0.018) in PCOS patients. Interatrial (19 ± 7.4 vs 15 ± 6.4 ms, *p* = 0.035) and intra-atrial [8.5 (1–19) vs 5 ms (1–20), *p* = 0.026] electromechanical delays were found to be significantly higher in PCOS patients.

**Conclusion:**

This study showed that patients with PCOS had increased inter- and intra-atrial conduction delays, and decreased LA passive emptying volumes and fractions.

## Background

Polycystic ovary syndrome (PCOS) is one of the most common endocrine/hormonal disorders that affect an estimated 5–10% of women of reproductive age. It is characterised by hyperandrogenism, chronic anovulation and polycystic ovaries.^1^ A high proportion of women with PCOS are associated with a higher than the normal incidence of insulin resistance, hyperlipidaemia and obesity, as well as cardiovascular disease.^2^

Gonadal steroids have an effect on cardiac ion currents and autonomic function and therefore may cause cardiac arrhythmias.^3^ About 65–75% of drug-induced ventricular arrhythmia occurs in women, whereas the incidence of atrial fibrillation or sudden death is lower than in men.^4^,^5^

It has recently been identified that left atrial (LA) volume and mechanical functional index are potential indicators of atrial arrhythmia and cardiac disease. LA function is an important factor for left ventricular (LV) filling, and atrial emptying pressure and volume may provide important information about LV resistance. As a result, the atrial emptying pattern is strongly affected by LV diastolic function. Intra- and interatrial conduction delay, which are evaluated by tissue Doppler imaging (TDI), as well as power Doppler (PD), are electrophysiological characteristics of the atria that are prone to atrial fibrillation (AF).^6^,^7^

Although there are some studies investigating QT dispersion and ventricular diastolic function in women with PCOS, there is no literature, to our knowledge, on the PD and electromechanical properties of patients with this disease. Therefore we aimed to investigate PD, inter- and intra-atrial conduction times and mechanical function of the LA in patients with PCOS.

## Methods

A total of 86 women were consecutively enrolled in this study. Forty-eight patients with PCOS (mean age 24 ± 4 years) and 38 normal healthy women as controls (mean age 27 ± 5 years), who attended the gynaecology clinic of Canakkale Onsekiz Mart University between March and May 2012, participated in this study.

PCOS patients were selected from subjects who were admitted due to oligo-amenorrhea, infertility or hirsutism. The control group was selected from healthy women who presented with infertility and the male factor was detected to be the cause of the infertility. PCOS was diagnosed by ultrasound if there were polycystic ovaries [enlarged ovaries (2–8 mm in diameter) with cysts ≥ 8], oligo-amenorrhea (intermenstrual interval > 35 days), hirsutism (Ferriman–Gallwey score ≥ 7) and elevated serum testosterone levels (≥ 2.7 nmol/l, convention factor 0.03467; 80 ng/dl).^8^

Patients with hypertension, diabetes mellitus, electrolyte imbalance, a history of chronic renal failure or a glomerular filtration rate < 60 ml/min according to the MDRD formula, chronic inflammatory disease, chronic lung disease, heart failure or valve disease, thyroid function disorders, history of arrhythmia, sleep-apnoea syndrome, smoking and drug use in the last three months were excluded. The study protocol was approved by the local ethics committee and written informed consent was obtained from all patients.

Laboratory, electrocardiographic and echocardiographic assessments were done on the second or third days of the menstrual cycle, which is the follicular phase. Fasting levels of blood glucose and insulin, lipid profiles and hormone levels were determined by standard laboratory methods. Insulin resistance as assessed using the homeostasis model assessment (HOMA–IR) calculation: fasting serum insulin (μIU/ml) × fasting plasma glucose (mg/dl)/405.^9^

## Analysis of electrocardiography

A 12-lead surface electrocardiogram was used to evaluate P-wave parameters. The paper speed was 50 mm/s and amplitude was 20 mm/mV. All electrocardiograms were recorded on the second or third day of the menstrual cycle. P waves were measured manually on all derivations and at least three cardiac cycles were recorded.

Pd was defined as the difference between the maximum (P_max_) and minimum (P_min_) P-wave duration. The onset of the P wave was defined as the point of first visible upward slope from baseline for positive waveforms, and as the point of first downward slope from baseline for negative waveforms. The return to baseline was considered as the end of the P wave.

## Echocardiography

Two-dimensional, M-mode, pulsed and colour-flow Doppler echocardiographic examinations were performed on all patients by one cardiologist on the second or third day of the menstrual cycle (Vivid 7 Pro, GE, Horten, Norway, 2–4 MHz phasedarray transducer). During echocardiography, a single-lead electrocardiogram was recorded simultaneously. Data were recorded from the average of three cardiac cycles.

M-mode and Doppler measurements were performed adhering to the American Society of Echocardiography guidelines.^10^ TDI was performed with transducer frequencies of 3.5–4 MHz. The monitor sweep was set at 100 mm/s. A pulsed Doppler sample volume was placed at the level of the LV septal mitral annulus, lateral mitral annulus and tricuspid annulus in the apical four-chamber view. Peak systolic, early diastolic (E) and late diastolic (A) velocities were obtained at these levels.

Atrial electromechanical coupling, the time interval from the onset of the P wave to the beginning of the late diastolic wave, was calculated from the lateral mitral annulus (PAlat), septal mitral annulus (PAsep) and tricuspid annulus (PAtri). Interatrial electromechanical delay was defined as the difference between PAlat and PAtri, and intra-atrial electromechanical delay was defined as the difference between PAsep and PAtri.^11^

LA volumes were measured echocardiographically by the biplane area–length method from the apical four-chamber view. LA maximal volume (V_max_) was calculated at the onset of mitral valve opening, LA minimum volume (V_min_) at the onset of mitral valve closure, and LA presystolic volume (Vp) at the beginning of the P wave on a surface ECG. LA passive emptying volume [(PEV) = V_max_ – Vp], LA passive emptying fraction [(PEF) = (V_max_ – Vp)/V_max_], LA active emptying volume [(AEV) = Vp – V_min_], LA active emptying fraction [(AEF) = (Vp – V_min_)/Vp], and LA total emptying volume [(TEV) = V_max_ – V_min_] were defined as LA emptying function parameters.^12^,^13^

## Statistical analysis

All continuous variables were expressed as mean ± standard deviation and median (interquartile range). All measurements were evaluated with the Kolmogorov–Smirnov and Shapiro–Wilk tests, and comparisons of parametric and non-parametric values between two groups were performed by means of the Mann–Whitney *U*-test or Student’s *t*-test. Univariate linear regression and stepwise multiple regression analyses were used to identify the clinical characteristics of interatrial electromechanical delay. Age, body mass index (BMI) and testosterone levels were entered into the model. All statistical studies were carried out with the SPPS program (version 15.0, SPSS, Chicago, Illinois, USA); *p* < 0.05 was accepted as statistically significant.

## Results

Clinical and laboratory findings of the subjects are shown in Table 1. Age and serum FSH levels, respectively, were significantly lower in patients with PCOS [24 ± 4 vs 30 ± 7 years, *p* < 0.01; and 5.07 (2.92–10.1) vs 7.68 (2.02–19.10) mIU/ml, *p* < 0.001]. BMI (22.5 ± 3.4 vs 25.4 ± 5.4 kg/m^2^, *p* = 0.029) and serum estradiol levels (28.8 ± 11.3 vs 43.2 ± 17.8 pg/ml, *p* < 0.001) were significantly higher in PCOS patients than in the control subjects.

**Table 1:** Patient characteristics and demographics

	*PCOS (n = 48)*	*Control (n = 38)*	*p-value*
Age (years)	24 ± 4	30 ± 7	0.001*
BMI (kg/m^2^)	25.4 ± 5.4	22.5 ± 3.4	0.029*
BSA (m^2^)	1.71 ± 0.18	1.64 ± 0.13	0.071
Waist/hip ratio	0.80 ± 0.05	0.77 ± 0.01	0.061
FSH (mIU/ml)	5.07 (2.92–10.1)	7.68 (2.02–19.10)	0.001*
LH (mIU/ml)	6.62 (2.35–39.25)	6.74 (2.03–19.47)	0.442
TSH (μIU /ml)	2.48 ± 2.36	1.78 ± 0.91	0.110
Estradiol (pg/ml)	43.2 ± 17.8	28.8 ± 11.3	0.001*
Testosterone (ng/dl)	75.5 (14.7–314)	17.2 (2.5–44)	0.001*
Fasting glucose (mg/dl)	86 ± 12	87 ± 8	0.945
Total cholesterol (mg/dl)	176 ± 34	181 ± 38	0.557
HDL (mg/dl)	53 ± 15	56 ± 14	0.399
LDL (mg/dl)	96 ± 28	101 ± 33	0.407
TG (mg/dl)	97 ± 74	87 ± 50	0.880
Fasting insulin (μIU /ml)	15.28 ± 23.45	12.74 ± 17.57	0.627
HOMA-IR	1.40 (0.37–36.15)	1.44 (0.38–18.99)	0.659
Heart rate (bpm)	82.02 ± 13.15	75.24 ± 11.02	0.014**
Systolic blood pressure	109 ± 7	105 ± 8	0.094
Diastolic blood pressure	70 ± 6	68 ± 8	0.227
P_max_ (msn)	97 ± 7	102 ± 8	0.013*
PM_min_ (msn)	70 ± 6	77 ± 7	0.001*
Pd (msn)	27 ± 5	24 ± 6	0.035*

PCOS = polycystic ovary syndrome; BMI = body mass index; BSA = body surface area; FSH = follicular stimulating hormone; LH = luteinising hormone; TSH = thyroid stimulating hormone; HDL = high-density lipoprotein; LDL= low-density lipoprotein; TG = triglycerides; HOMAIR = homeostasis model assessment of insulin resistance; P_max_ = maximum P-wave duration; P_min_ = minimum P-wave duration; Pd = P-wave dispersion; *Mann–Whitney _U_-test; **Independent _t_-test.

Serum testosterone levels were higher in patients with PCOS than in the control group [75.5 (14.7–314) vs 17.2 (2.5–44) ng/dl, *p* < 0.001]. Heart rate (82.02 ± 13.15 vs 74.24 ± 11.02 bpm, *p* = 0.014) and Pd were significantly increased in PCOS patients (27 ± 5 vs 24 ± 6 ms).

Echocardiographic findings of the study population are given in Table 2. LV diastolic and systolic diameters, ejection fraction, fractional shortening, LA diameters, and E and A waves were similar in both groups. The transmitral E/A ratio was significantly lower in PCOS patients than in the controls (1.5 ± 0.3 vs 1.7 ± 0.4, *p* = 0.023). The peak systolic myocardial velocity was higher in patients with PCOS (0.09 ± 0.01 vs 0.08 ± 0.01 m/s, *p* = 0.02). The myocardial early diastolic wave (E’) and E/E’ ratio were similar in both groups.

**Table 2:** Echocardiographic properties of the study population

	*PCOS (n = 48)*	*Controls (n = 38)*	*p-value*
LVDD (mm)	44.3 ± 3.7	43.3 ± 3.6	0.222
LVSD (mm)	27.7 ± 3.3	26.9 ± 4.2	0.330
LVEF (%)	68 ± 4	69 ± 3	0.433
LA (mm)	33 ± 4	32 ± 3	0.171
Mitral E wave (cm/s)	91.7 ± 15.9	94.4 ± 19	0.483
Mitral A wave (cm/s)	59.9 ± 12.8	55 ± 12.4	0.085
E/A	1.5 ± 0.3	1.7 ± 0.4	0.023*
DT (ms)	173 ± 16	180 ± 19	0.114
Peak S (m/s)	0.09 ± 0.01	0.08 ± 0.01	0.02**
LV E′ (cm/s)	14.5 ± 3.5	13.5 ± 2.9	0.190
E/E′	6.5 ± 1.6	7.1 ± 1.8	0.116
LV IVRT (ms)	112 ± 15	109 ± 15	0.282
LV IVCT (ms)	371 ± 35	373 ± 36	0.790

PCOS = polycystic ovary syndrome; LV = left ventricle; DD = diastolic diameter; SD = systolic diameter; EF = ejection fraction; FS = fractional shortening, LA = left atrium, DT = deceleration time, LV E′ = left ventricle early diastolic velocity, IVRT = ısovolumetric relaxation time; IVCT = isovolumetric contraction time; *Independent *t*-test; **Mann–Whitney *U*-test.

There were no differences in LA V_max_, LA V_min_, and Vp between the groups. The LA active emptying volume and active emptying fraction were similar. The passive emptying volume (12.54 ± 4.39 vs 15.28 ± 3.85 ml/m^2^, *p* = 0.004) and passive emptying fraction [54.4 (21–69) vs 59.1 (28–74)%, *p* = 0.008] were significantly decreased in PCOS patients. The total emptying volume was significantly decreased (17.9 ± 5.49 vs 20.67 ± 4.29 ml/m^2^, *p* = 0.018) in PCOS patients (Table 3).

**Table 3:** Atrial conduction times and left atrial measurements of the study population

	PCOS (n=48)	Controls(n=38)	p-value
PA lateral (ms)	57 (34–87)	43 (34–77)	0.001*
PA septal (ms)	48 (24–68)	34 (25–67)	0.001*
PA tricuspid (ms)	39 (21–60)	28 (22–65)	0.001*
PA lateral tricuspid (ms)	19 ± 7.4	15.6 ± 6.4	0.035**
PA septal tricuspid (ms)	8.50 (1–19)	5 (1–20)	0.026*
LA V_max_ (ml/m^2^)	24.59 ± 6.7	26.31 ± 4.95	0.201
LA Vmin (ml/m^2^)	6.11 (2.41–19.23)	5.60 (2.33–11.49)	0.164
LA Vp (ml/m^2^)	11.06 (6.01–29.23)	10.65 (5.23–20.53)	0.398
LA PEV (ml/m^2^)	12.54 ± 4.39	15.28 ± 3.85	0.004**
LA PEF (%)	54.4 (21–69)	59.1 (28–74)	0.008**
LA AEV (ml/m^2^)	5.45 ± 2.18	5.39 ± 2.12	0.903
LA AEF (%)	45.9 ± 10.7	48.7 ± 10.5	0.252
LA TEV (ml/m^2^)	17.9 ± 5.49	20.67 ± 4.29	0.018**

PA = time interval from the onset of P wave to the beginning of the late myocardial diastolic velocity, LA = left atrium; V_max_ = maximum volume; V_min_ = minimum volume; Vp = volume of the beginning atrial systole; PEV = passive emptying volume; PEF = passive emptying fraction; AEV = active emptying volume; AEF = active emptying fraction; TEV = total emptying volume; *Mann–Whitney *U*-test; **Independent *t*-test.

Tissue Doppler echocardiography measurements are shown in Table 3. PAlat [57 (34–87) vs 43 (34–77) ms, *p* < 0.01], PAsep [48 (42–68) vs 34 (25–67) ms, *p* < 0.01] and PAtri [39 (21–60) vs 28 (22–65) ms, *p* = 0.001] were significantly longer in patients with PCOS. Interatrial and intra-atrial electromechanical delays were found to be significantly higher in PCOS patients. Values of PAlat – PAtri were 19 ± 7.4 and 15 ± 6.4 ms in PCOS patients and control subjects, respectively (*p* = 0.035). Median values of PAsep – PAtri were 8.5 (1–19) and 5 (1–20) ms (*p* = 0.026), respectively.

Age and serum testosterone levels were associated with interatrial electromechanical delay in the linear regression analysis (*p* = 0.071, β = 0.201 and *p* = 0.052, β = 0.242, respectively). In a multivariate stepwise regression analysis, age was demonstrated to be an independent predictor of interatrial electromechanical delay (*p* = 0.013, β = –0.321) (Table 4).

**Table 4:** Relation between interatrial conduction delay and clinical findings. Multivariate analysis model included age, BMI and testosterone level

	Univariate		Multivariate	
	β	p	β	p
Age	0.201	0.071	–0.321	0.013
BMI	0.031	0.802		
Estradiol	0.160	0.160		
Insulin	0.047	0.716		
HOMA-IR	0.037	0.779		
Testosterone	0.242	0.052		
E/A ratio	–0.010	0.930		
LA passive fraction	–0.063	0.584		
Heart rate	0.034	0.761		
Total emptying volume	0.020	0.861		
LA diameter	0.130			

## Discussion

In the present study, we showed that Pd, and inter- and intraatrial conduction times were increased, left atrial mechanical function was impaired, and transmitral E/A ratio was decreased in patients with PCOS. To our knowledge, this is the first study that has assessed the mechanical and electromechanical functions of the LA in patients with PCOS.

Previous investigations suggested that endothelial and diastolic dysfunction occurs in patients with PCOS due to insulin resistance and high androgen levels.^14^,^15^ Diastolic dysfunction is considered to be an early sign of coronary artery disease. Accordingly, Yarali *et al*. reported that PCOS patients had a lower peak E velocity and E/A ratio.^16^

A study showed a negative correlation between insulin levels and E/A ratio, which suggests that PCOS patients may be more prone to develop diastolic dysfunction.^17^ Another study showed that LV ejection fraction, transmitral E and A waves, E/A ratio, deceleration time, isovolumetric relaxation time and tissue Doppler parameters were not significantly different between patients with PCOS and control subjects.^18^ In our study, LV systolic function and transmitral E and A waves were similar in the two groups, but E/A ratio was decreased in the study patients, suggesting impaired diastolic function.

Atrial fibrillation is the most commonly observed arrhythmia in clinical practice and is associated with cardiovascular morbidity and mortality.^19^-^21^ Identifying the risk factors that generate AF is important. It was thought that enlargement and increased pressure of the LA have an effect on P-wave disturbances.^22^-^24^ The presence of P-wave dispersion, and interand intra-atrial conduction delays mean that sinus impulses have inhomogeneous propagation. This is a well-known electrocardiographic characteristic of atria that are prone to AF.^6^,^25^

The autonomic nervous system plays an important role in heart rate and the conduction system. Imbalance between the sympathetic nervous system and vagal tone is an important predictor of AF.^26^,^27^

Gonadal sex hormones affect heart rate and atrioventricular conduction time. Fulop *et al*. reported that heart rate is moderately reduced after surgical castration in both male and female canines. Heart rate is increased and PQ interval is lengthened with oestrogen replacement in male canines, and with testosterone replacement in female canines. Therefore, oestrogen and testosterone have identical effects on the heart rate and atrioventricular conduction time.^3^

In our study, Pd, inter- and intra-atrial conduction times were increased in patients with PCOS. Therefore resting heart rate was higher in these patients. Elevated serum levels of oestrogen and testosterone may explain these findings.

Ventricular filling pressure is an indicator of LV diastolic function, and LA function is an important determinant of LV diastolic filling.28 LA passive emptying volume is related to increased LV end-diastolic pressure.^12^ We found that LA mechanical function was impaired in PCOS patients.

Insulin resistance, obesity and hyperlipidaemia, risk factors for cardiovascular disease, are more frequent in patients with PCOS.^2^ Studies showed that arterial stiffness, endothelial dysfunction and LV diastolic dysfunction were increased in obese patients with PCOS.^29^,^30^ Additionally, diastolic function may worsen with age. In our study E/A ratio was lower in women with PCOS, even though the PCOS patients were significantly younger. BMI differences were insignificant between the groups.

Impairment of LA mechanical and electromechanical function is known to be a risk factor for AF. LA mechanical function, conduction times and Pd have not been evaluated previously in patients with PCOS. Although there were no known cardiac risk factors, obesity or insulin resistance, we found not only impaired LV diastolic parameters but also increased LA conduction times and Pd in these patients. Hence, we suggest that decreased LA mechanical function, lengthening inter- and intra-atrial conduction times, and increased Pd may occur before the appearance of cardiovascular risk factors such as hypertension, diabetes mellitus and hyperlipidaemia.

Age is an important risk factor for the development of AF.^19^ However Turhan *et al*. reported a positive correlation between age and Pd, and LV diastolic parameters.^31^ In our study, although the patients with PCOS were younger, they still had significant impairment in Pd, LA mechanical dysfunction, and increased electromechanical delay.

Our study has some limitations that need to be addressed. It was based on a relatively small group of patients and the patients with PCOS were younger than the controls. There is a need for longitudinal studies to follow up on subjects and controls.

## Conclusion

This study showed that patients with PCOS had increased inter- and intra-atrial conduction delays, decreased LA passive emptying volume and fraction, and lower E/A ratios. Increased Pd is a risk factor for developing AF, therefore PCOS patients may have a high risk for developing atrial arrhythmias, unless they have other traditional cardiovascular risk factors.
